# The association between body dysmorphic symptoms and suicidality among adolescents and young adults: a genetically informative study

**DOI:** 10.1017/S0033291720002998

**Published:** 2022-05

**Authors:** Georgina Krebs, Lorena Fernández de la Cruz, Frühling V. Rijsdijk, Daniel Rautio, Jesper Enander, Christian Rück, Paul Lichtenstein, Sebastian Lundström, Henrik Larsson, Thalia C. Eley, David Mataix-Cols

**Affiliations:** 1 King's College London, MRC Social, Genetic and Developmental Psychiatry Centre, Institute of Psychiatry, Psychology & Neuroscience, London, UK; 2 National and Specialist OCD and Related Disorders Clinic for Young People, South London, UK; 3 Maudsley NHS Foundation Trust, London, UK; 4 Department of Clinical Neuroscience, Centre for Psychiatry Research, Karolinska Institutet, & Stockholm Health Care Services, Stockholm County Council, Stockholm, Sweden; 5 Department of Medical Epidemiology and Biostatistics, Karolinska Institutet, Stockholm, Sweden; 6 Gillberg Neuropsychiatry Centre, Centre for Ethics, Law and Mental Healt, University of Gothenburg, Gothenburg, Sweden; 7 School of Medical Sciences, Örebro University, Örebro, Sweden

**Keywords:** adolescence, body dysmorphic disorder, genetics, suicidal ideation, suicide attempts, twin design

## Abstract

**Background:**

Previous research indicates that body dysmorphic disorder (BDD) is associated with risk of suicidality. However, studies have relied on small and/or specialist samples and largely focussed on adults, despite these difficulties commonly emerging in youth. Furthermore, the aetiology of the relationship remains unknown.

**Methods:**

Two independent twin samples were identified through the Child and Adolescent Twin Study in Sweden, at ages 18 (*N* = 6027) and 24 (*N* = 3454). Participants completed a self-report measure of BDD symptom severity. Young people and parents completed items assessing suicidal ideation/behaviours. Logistic regression models tested the association of suicidality outcomes with: (a) probable BDD, classified using an empirically derived cut-off; and (b) continuous scores of BDD symptoms. Bivariate genetic models examined the aetiology of the association between BDD symptoms and suicidality at both ages.

**Results:**

Suicidal ideation and behaviours were common among those with probable BDD at both ages. BDD symptoms, measured continuously, were linked with all aspects of suicidality, and associations generally remained significant after adjusting for depressive and anxiety symptoms. Genetic factors accounted for most of the covariance between BDD symptoms and suicidality (72.9 and 77.7% at ages 18 and 24, respectively), but with significant non-shared environmental influences (27.1 and 22.3% at ages 18 and 24, respectively).

**Conclusions:**

BDD symptoms are associated with a substantial risk of suicidal ideation and behaviours in late adolescence and early adulthood. This relationship is largely explained by common genetic liability, but non-shared environmental effects are also significant and could provide opportunities for prevention among those at high-risk.

## Introduction

Body dysmorphic disorder (BDD) is characterised by a distressing and impairing preoccupation with perceived or slight defects in physical appearance, typically accompanied by time-consuming, repetitive behaviours (American Psychiatric Association, 2013; World Health Organization, 2018). The disorder affects approximately 2% of adolescents and adults (Veale, Gledhill, Christodoulou, & Hodsoll, 2016), and confers substantial morbidity, including reduced quality of life, poor social and occupational functioning, and high levels of comorbid psychopathology (Didie, Menard, Stern, & Phillips, 2008; Phillips, Menard, Fay, & Pagano, 2005). Preliminary evidence suggests that BDD is also associated with alarmingly high rates of suicidality. A meta-analysis found the weighted pooled rate of suicidal ideation and suicide attempts in BDD patients to be 53 and 24%, respectively (Angelakis, Gooding, & Panagioti, 2016). These rates represent approximately a four-fold increased risk for suicidal ideation and three-fold increased risk for suicide attempts among BDD sufferers, relative to controls (Angelakis et al., 2016). However, most previous studies of BDD and suicidality have involved specialist clinical or study cohorts and may be subject to selection bias. Only a small number of studies have examined the association of BDD with suicidality in community samples, and these have involved modest sample sizes (308–2552 individuals) and a small number of BDD cases (11–62 individuals) (Buhlmann et al., 2010; Möllmann, Dietel, Hunger, & Buhlmann, 2017; Rief, Buhlmann, Wilhelm, Borkenhagen, & Brähler, 2006; Schieber, Kollei, de Zwaan, & Martin, 2015). Additionally, only two studies focussed on young people (Albertini & Phillips, 1999; Dyl, Kittler, Phillips, & Hunt, 2006), despite the fact that BDD usually emerges during adolescence and adolescent-onset BDD has been reported to be associated with higher rates of suicidality (Bjornsson et al., 2013; Phillips et al., 2006). Moreover, suicide is a major public health concern for adolescents and young adults, ranked as the second leading cause of death for 15–29-year-olds globally (Centers for Disease Control and Prevention, 2017; Patton et al., 2009; World Health Organization, 2014). Thus, there is a need for large-scale, community-based studies to establish the true link between BDD and suicidality, particularly among young people.

A further question is: what factors underpin the relationship between BDD and suicidality? Understanding the aetiology of this association could have important clinical implications, informing the development of more effective strategies for identifying and reducing the risk of suicidality in BDD. One possibility is that the association between BDD and suicidality is predominantly mediated by environmental factors. For example, suicidality could arise as a result of the emotional and psychosocial burden of BDD and/or both phenotypes could have common environmental risks, such as peer victimisation (Baldwin et al., 2019; Geoffroy et al., 2016; Lavell, Webb, Zimmer-Gembeck, & Farrell, 2018; Webb et al., 2015). Alternatively, the association between BDD and suicidality could largely reflect common genetic influences. Twin studies have indicated that BDD is probably heritable, with genetic influences accounting for 37–49% of the variance in body dysmorphic symptoms (Enander et al., 2018; Monzani et al., 2012a). Similarly, genetic factors have been estimated to explain 41–74% and 30–55% of the variance in suicidal ideation and attempts, respectively (Althoff et al., 2012; Fu et al., 2002; Maciejewski et al., 2014; Statham et al., 1998). It is, therefore, possible that BDD and suicidality share common genetic risks, consistent with recent findings in obsessive-compulsive disorder (OCD), a closely related phenotype (Sidorchuk et al., in press).

The current study used a genetically-informative design to explore the relationship between body dysmorphic symptoms and suicidality in a large, population-based sample of adolescents and young adults. Based on previous literature, we hypothesised that BDD symptoms would be significantly associated with suicidality. Additionally, we expected that this association would be attenuated, but not fully accounted for, by co-occurring symptoms of depression and anxiety, given their known link with both BDD symptoms (Gunstad & Phillips, 2003; Möllmann et al., 2017; Schneider, Turner, Mond, & Hudson, 2017) and suicidality (Hawgood & De Leo, 2008; Sareen et al., 2005). We further hypothesised that the relationship between BDD symptoms and suicidality would be explained by genetic and non-shared environmental influences, in line with previous research in OCD (Sidorchuk et al., in press).

## Methods

### Study population

The current study used participant data from the Child and Adolescent Twin Study in Sweden (CATSS; Anckarsäter et al., 2011), a longitudinal study of all twins born in Sweden since July 1992. The present analyses focussed on data obtained when the twins were 18- (CATSS-18) and 24-years-old (CATSS-24). At both time points, twins completed a battery of questionnaires, including a measure of BDD symptoms and several items relating to suicidality. At age 18, parents/caregivers also reported on suicidality in their child. BDD symptom and suicidality data were available for 6027 participants in CATSS-18 and 3454 participants in CATSS-24. No participants had relevant data available at both ages since the BDD measure was only introduced in 2013. At the point at which the current study was started, the CATSS-18 participants with DCQ data had not yet reached 24 years of age. Therefore, the 18- and 24-year-olds in the current study represent independent samples. Demographic and clinical characteristics for both cohorts are shown in Table 1.

**Table 1.** Demographic and clinical characteristics of participants at age 18 (CATSS-18) and age 24 (CATSS-24).

	Cohort	
	CATSS-18 (*N* = 6027)	CATSS-24 (*N* = 3454)
Sex, *n* (%)		
Male	2532 (42.0%)	1482 (42.9%)
Female	3495 (58.0%)	1972 (57.1%)
Zygosity, *n* (%)		
Monozygotic	1664 (27.6%)	1112 (32.2%)
Dizygotic	4115 (68.3%)	2238 (64.8%)
Unknown	248 (4.1%)	104 (3.0%)
DCQ total, mean (s.d.)	3.82 (4.39)	3.94 (4.01)
SCARED total, mean (s.d.	15.91 (11.50)	–
CES-D total, mean (s.d.)	8.41 (5.93)	–
HADS-anxiety, mean (s.d.)	–	6.65 (3.82)
HADS-depression, mean (s.d.)	–	5.27 (3.56)
Suicidality composite, *n* (%)		
⩾ 1 symptom of suicidality endorsed	257 (6.6%)	863 (25.1%)
No symptom of suicidality endorsed	3649 (93.4%)	2579 (74.9%)

DCQ, Dysmorphic Concerns Questionnaire; SCARED, Screen for Child Anxiety Related Emotional Disorders; CES-D, Center for Epidemiologic Studies Depression Scale; HADS, Hospital Anxiety and Depression Scale.

Twin zygosity was determined by a panel of 48 single-nucleotide polymorphisms (SNPs) derived for zygosity analyses (Hannelius et al., 2007). If DNA was unavailable, an algorithm based on five questions of twin similarity was used, as previously described (Anckarsäter et al., 2011). Ethical approval for CATSS was granted by the Stockholm Regional Ethics Review Board (CATSS-18: reference number 2010/1410-31/1; CATSS-24: reference number 2015/1947-31/4).

### Measures

#### Body dysmorphic disorder

The Dysmorphic Concern Questionnaire (DCQ; Oosthuizen, Lambert, and Castle, 1998) is a seven-item, self-report measure that assesses the extent to which individuals are concerned about their physical appearance or body malfunction (e.g. excessive body odour, flatulence, sweating). Items are rated on a 4-point scale ranging from 0 (none) to 3 (much more than most people), yielding a total score between 0 and 21. The DCQ has been shown to have good internal consistency and construct validity (Jorgensen, Castle, Roberts, & Groth-Marnat, 2001; Mancuso, Knoesen, & Castle, 2010; Oosthuizen et al., 1998; Schieber, Kollei, de Zwaan, & Martin, 2018), and has a single factor structure (Enander et al., 2018; Jorgensen et al., 2001; Monzani et al., 2012a; Oosthuizen et al., 1998). In the current study, the DCQ demonstrated good internal consistency (Cronbach's *α* = 0.87 both at ages 18 and 24).

In the present study, the DCQ instructions were modified to explicitly state that the responder should not include concerns relating to ‘weight or being too fat’, in order to ensure that the measure captured BDD symptoms rather than eating disorder psychopathology. In addition, a supplementary question was included to assess for genuine physical abnormalities or disfigurements. Participants who provided unambiguous reports of physical abnormalities in appearance (e.g. amputated leg, cleft palate, supernumerary fingers) were excluded from the analyses (age 18: *n* = 15, 0.2%; age 24: *n* = 9, 0.3%).

Scores on the DCQ were used both categorically, to define cases of ‘probable BDD’, and continuously. A number of empirically derived cut-offs have been proposed for the DCQ (Enander et al., 2018; Mancuso et al., 2010; Monzani et al., 2012a). We selected the most conservative cut-off ⩾17 to classify probable BDD. This cut-off has been shown to correctly identify 96% of diagnosed BDD patients, with a sensitivity of 56% and a specificity of 99% (Enander et al., 2018).

#### Suicidality

Suicidal ideation and behaviours were assessed using a range of questionnaire items (see online Supplementary Table S1 for details). To assess suicidal ideation at age 18, parents were asked whether their child talked about killing themselves. To assess suicide attempts at this age, parents were asked whether their child had deliberately harmed themselves or attempted suicide and young people were asked whether they had ever deliberately attempted to kill themselves. At age 24, participants were asked whether they wished that they were dead (thoughts of death) and if they had ever had thoughts of taking their life (suicidal ideation). To assess suicide attempts, participants were asked whether they had ever attempted to take their life. This was further qualified by asking if suicide attempts had required medical attention or lead to hospital admission. All items were coded with binary responses (yes/no). At age 24, a suicidality composite was created by combining the suicidal ideation and suicide attempt items. These two items were selected because they closely corresponded to established definitions of suicidal ideation and attempts (Silverman, Berman, Sanddal, O'Carroll, & Joiner, 2007). The composite was coded as a binary outcome (yes/no). Thus, an individual scored positive on the suicidality composite if they had endorsed either of the constituent items. Of note, a suicidality composite was not created at age 18 because only one self-report item was available. Self- and parent-reported items were not combined since (a) parents typically underreport suicidality (Breton, Tousignant, Bergeron, & Berthiaume, 2002); and (b) the parent-reported items did not correspond as well to recognised definitions of suicidal ideation and attempts (Silverman et al., 2007).

#### Depression and anxiety

At age 18, depressive symptoms were assessed using the Iowa version of the Center for Epidemiologic Studies Depression Scale (CES-D; Kohout, Berkman, Evans, & Cornoni-Huntley, 1993; Radloff, 1977). This self-report measure comprises 11 items scored on a 4-point scale ranging from 0 (rarely or none of the time) to 3 (most or all of the time), giving a total score between 0 and 33. The scale has good psychometric properties and correlates highly with the original CES-D (Carpenter et al., 1998). In the current study, internal consistency for the CES-D was good (Cronbach's *α* = 0.87).

Anxiety was assessed at age 18 using the 38-item, self-report version of Screen for Child Anxiety Related Emotional Disorders (SCARED; Birmaher et al., 1997). Items are scored on a 3-point scale ranging from 0 (almost never true) to 2 (true most of the time), yielding a total score ranging from 0 to 76. The measure has good psychometric properties (Birmaher et al., 1997; Hale, Raaijmakers, Muris, & Meeus, 2005; Monga et al., 2000). Internal consistency in the current sample was excellent (Cronbach's *α* = 0.93).

At age 24, anxiety and depressive symptoms were assessed using the Hospital Anxiety and Depression Scale (HADS; Zigmond and Snaith, 1983). The HADS is a 14-item self-report measure, comprising a 7-item general anxiety subscale (HADS-A) and a 7-item depression subscale (HADS-D). Items are scored on a 4-point scale ranging from 0 (never) to 3 (almost always). The HADS is psychometrically robust (Bjelland, Dahl, Haug, & Neckelmann, 2002) and, in the current study, internal consistency was good (Cronbach's *α* = 0.83 and 0.79 for HADS-A and HADS-D, respectively).

### Statistical analysis

#### Phenotypic analyses

Associations of suicidal ideation and behaviours (binary variables) with BDD symptoms were examined in a series of logistic regressions. We tested the association of (a) probable BDD (total DCQ score ⩾17); and (b) BDD symptoms (total DCQ score) with suicidality variables at ages 18 and 24. The first set of analyses were more clinically relevant, while the second allowed us to maximise statistical power. In the second set of analyses, we examined the association of BDD symptoms with suicidality: without adjustment for comorbidity; with adjustment for depressive symptoms; and with adjustment for both depressive and anxiety symptoms. This stepwise approach enabled us to separately gauge the extent to which depressive and anxiety symptoms accounted for the association between BDD symptoms and suicidality, which was important since anxiety can be viewed as a core part of the BDD phenotype. Logistic regressions were conducted in STATA version 14.2, using the robust cluster option to account for non-independence of twins/siblings. The DCQ and SCARED showed evidence of positive skew and were therefore log-transformed prior to analyses (see online Supplementary Table S2 for the skewness of variables). In addition, continuous variables were standardised for ease of comparison across scales. All regression models controlled for age and sex.

#### Genetic analyses

The aetiology of the associations between BDD symptoms and suicidality was explored using bivariate models. The twin design compares the degree of phenotypic similarity between MZ twins, who share 100% of their genes, with DZ twins, who share on average 50% of their segregating genes (Rijsdijk & Sham, 2002). Within-pair correlations for MZ twins are compared with those for DZ twins. Greater MZ compared to DZ phenotypic similarity is attributed to additive genetic effects (A). Within-pair similarity that is not accounted for by genetic factors is attributed to shared environmental effects (C). Within-pair differences between MZ twins are attributed to non-shared environmental effects (E), defined as non-genetic factors that give rise to phenotypic differences between siblings. This estimate also includes measurement error. The same principles can be extended to multivariate twin models, in order to estimate the aetiology of associations between variables. Bivariate models are based on cross-twin cross-trait correlations (e.g. the correlation between twin 1's score on the first trait and twin 2's score on the second trait). A higher cross-twin cross-trait correlation for MZ compared to DZ twins indicates that genetic factors have a degree of influence on the phenotypic covariance of two traits.

Bivariate correlated factor models were used to decompose the phenotypic association of BDD symptoms with suicidality into genetic and environmental influences. At age 18, the self-reported suicide attempt item was selected for analysis since it corresponded best to established definitions of suicidality (Silverman et al., 2007). At age 24, the suicide composite was selected for the primary analysis in order to maximise power, but self-reported suicidal ideation and suicide attempts were also examined separately in subsequent sensitivity analyses. At both ages, BDD symptoms were modelled as a continuous variable (i.e. total DCQ score). Suicidality variables were coded as binary outcomes and were therefore modelled using liability thresholds. Liability threshold models assume that ordered categories (in this case, the presence or absence of suicidality) reflect an imprecise measurement of an underlying normal distribution of liability, with one or more threshold discriminating between the different categories (Rijsdijk & Sham, 2002).

The relative fit of ACE models, with and without sex differences, were compared. Quantitative and qualitative sex differences were tested to see whether males and females differ in magnitude or nature of genetic and environmental influences, respectively. Scalar models were tested to assess whether differences in BDD symptoms between males and females were due to variance differences rather than differences in underlying genetic and environmental influences. Genetic models were compared with each other and with a constrained correlation model (hereafter referred to as a saturated model) in which means, variances, and thresholds were equated across twin order and zygosity groups for males and females.

Genetic modelling was conducted within R using OpenMx (Boker et al., 2011). BDD symptoms were age and sex regressed prior to analysis and as is standard in twin modelling to avoid artificial inflation of MZ *v.* DZ correlations (McGue & Bouchard, 1984). Models were fitted using raw data full information maximum likelihood. The main fit statistic provided by OpenMx for raw data modelling is minus twice the log-likelihood (−2LL) of the observations, which provides a relative measure of fit, since differences in −2LL are Chi-square distributed. We also examined model fit using Akaike information criterion (AIC), with lower values indicating a better balance between explanatory power and parsimony. A difference in AIC ⩾3 indicates support for the lower AIC model (Burnham & Anderson, 1998). Significance of parameters is established by 95% maximum likelihood confidence intervals (CI).

## Results

### Phenotypic findings

According to both self- and parent-report, individuals with probable BDD were significantly more likely to experience suicidal ideation and suicidal behaviours at ages 18 and 24, relative to those without (see Table 2). Approximately a quarter of individuals with probable BDD self-reported that they had attempted suicide at some point during their lifetime (ages 18 and 24), of whom two-thirds had sought medical help and nearly half had been hospitalised (age 24). Parent-reported rates were lower, for both current suicidal ideation and suicide attempts (age 18). Self-reported lifetime suicidal ideation was the most commonly endorsed item, reported by approximately two-thirds of those with probable BDD (age 24).

**Table 2.** Rates of suicidality in participants with and without probable body dysmorphic disorder.

	Probable BDD group^a^	Non-BDD group	Odds ratio
Age 18			
Suicide attempts (self-report)	27.7 (20.3–36.6)	6.0 (5.4–6.6)	5.08 (3.31–7.79)***
Suicidal ideation (parent-report)	12.7 (6.6–22.9)	1.6 (1.2–2.0)	6.01 (2.78–12.95)***
Suicide attempts (parent-report)	11.1 (5.6–21.0)	1.4 (1.0–1.8)	7.45 (3.63–15.29)***
Age 24			
Desire to be dead (self-report)	24.6 (15.2–37.3)	3.1 (2.6–3.8)	9.39 (5.13–17.18)***
Suicidal ideation (self-report)	65.6 (52.5–76.6)	23.3 (21.9–24.7)	6.15 (3.57–10.57)***
Suicide attempt (self-report)	24.6 (15.2–37.3)	4.9 (4.2–5.7)	5.64 (3.12–10.18)***
Suicide attempt requiring medical attention (self-report)	16.7 (9.0–28.7)	2.5 (2.1–3.1)	7.91 (3.69–16.98)***
Suicide attempt requiring hospital admission (self-report)	10.0 (4.5–20.9)	1.5 (1.1–1.9)	6.70 (2.63–17.07)***

BDD, body dysmorphic disorder.

*Note:* Proportions are presented as percentages. 95% confidence intervals are shown in parentheses.

a Clinically significant body dysmorphic symptoms defined as a score ⩾17 on the Dysmorphic Concern Questionnaire (DCQ). At age 18, 2.0% (*n* = 120) of the sample scored above and 98.0% (*n* = 5893) scored below the cut-off for probable BDD. At age 24, 1.8% (*n* = 61) of the sample scored above and 98.2% (*n* = 3392) scored below the cut-off for probable BDD. Sample size for regression models ranged from 3917 to 6013 at age 18, and from 3435 to 3453 at age 24.

*p < 0. 05; **p < 0.01; ***p < 0.001.

Table 3 shows the results of logistic regression models testing the association of continuous scores of BDD symptoms with suicidal ideation and suicide behaviours, with and without adjustment for coexisting symptoms of depression and anxiety. In the unadjusted models, BDD symptoms were significantly associated with all suicidality outcomes at ages 18 and 24. When adjusting for depression and anxiety, the strength of the associations reduced but nevertheless remained significant for all suicidality outcomes, except parent-reported suicide attempts at age 18.

**Table 3.** Results of logistic regression models testing the association between body dysmorphic symptoms and suicidality.

	Odds ratios		
	Without adjustment for depression and anxiety	With adjustment for depression	With adjustment for depression and anxiety
Age 18			
Suicide attempts (self-report)	2.04 (1.80–2.31)***	1.33 (1.16–1.53)***	1.25 (1.08–1.44)**
Suicidal ideation (parent-report)	2.56 (1.88–3.49)***	1.49 (1.07–2.08)*	1.55 (1.12–2.13)**
Suicide attempts (parent-report)	2.27 (1.63–3.16)***	1.20 (0.86–1.68)	1.20 (0.83–1.73)
Age 24			
Desire to be dead (self-report)	2.93 (2.21–3.89)***	1.81 (1.35–2.43)***	1.43 (1.05–1.94)*
Suicidal ideation (self-report)	2.14 (1.94–2.37)***	1.79 (1.62–1.98)***	1.59 (1.43–1.76)***
Suicide attempt (self-report)	2.29 (1.88–2.78)***	1.85 (1.51–2.26)***	1.54 (1.26–1.88)***
Suicide attempt requiring medical attention (self-report)	2.31 (1.77–3.02)***	1.87 (1.42–2.46)***	1.56 (1.18–2.06)**
Suicide attempt requiring hospital admission (self-report)	2.41 (1.67–3.47)***	1.84 (1.27–2.68)**	1.54 (1.06–2.25)*
Suicidality composite	2.18 (1.97–2.40)***	1.81 (1.64–2.00)***	1.61 (1.45–1.78)***

*Note:* Predictors (BDD, depression, and anxiety) were all modelled as continuous variables. Sample size for analyses ranged from 3655 to 6021 at age 18, and from 3379 to 3454 at age 24. All regression models controlled for age, sex, and relatedness of twin members using robust clustering. 95% confidence intervals are shown in parentheses. *p < 0. 05; ** *p* < 0.01; ***p < 0.001

### Genetic findings

The aetiology of the association between BDD symptoms and suicidality was examined using bivariate correlated factors models. Fit statistics are shown in online Supplementary Tables S3 and S4. At both ages 18 and 24, the scalar model, which allows for variance differences between the sexes, provided a better fit than the no sex differences model. This indicates variance differences in BDD symptoms between males and females. The scalar ACE models indicated no significant influence of C (shared environment) on either BDD symptoms or suicidality at age 18 or 24, with estimates close to zero. We, therefore, tested a more parsimonious scalar AE model. Although the quantitative sex differences ACE model provided a slightly better fit at both ages, the loss of statistical power (due to smaller sub-samples of males and females) resulted in imprecise estimates which could not be interpreted. The scalar AE model was therefore selected as the final model and was not a significantly poorer fit than the saturated model or the quantitative sex difference model at either age.

**Fig. 1.**
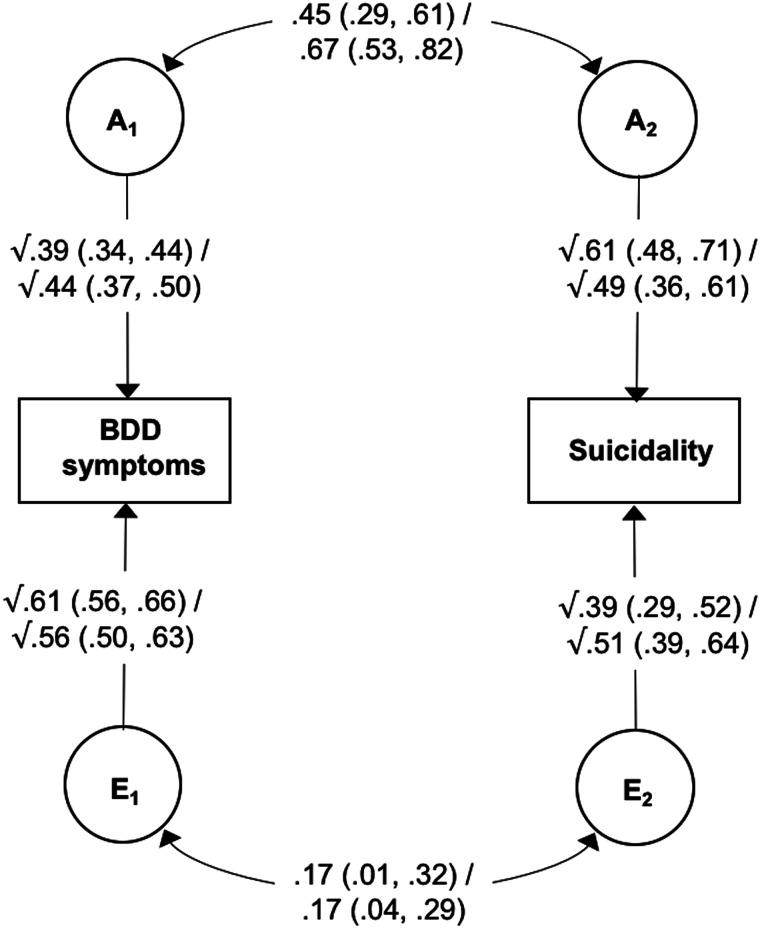
Bivariate correlated factor model showing genetic and non-shared environmental influences on body dysmorphic symptoms and suicide attempts at age 18/suicidality at age 24. *Note:* BDD, body dysmorphic disorder; A, additive genetic effects; E, non-shared environmental effects. Values on single-headed arrows are standardised path estimates; values on double-headed arrows are correlation coefficients; 95% confidence intervals are shown in parentheses. Path estimates can be used to calculate the proportion of the covariance between the two phenotypes that is accounted for A and E. For example, the genetic contribution to the association between BDD symptoms and suicidality at age 18 can be calculated by tracing the path between these two variables via A (√0.39 × 0.45 × √0.61) and dividing it by the combination of the paths between BDD symptoms and suicidality via A and E (√0.39 × 0.45 × √0.61 + √0.61 × 0.17 × √0.39).

Model estimates at ages 18 and 24 are shown in Fig. 1. The heritability of BDD symptoms was estimated to be 39% at age 18 and 44% at age 24, with the remaining variance accounted for by non-shared environmental factors. The heritability of suicide attempts at age 18 was estimated to be 61%, while the heritability of suicidality at age 24 was estimated to be 49%, with non-shared environmental influences accounting for the remaining variance at both ages. Of particular relevance to the current study, results showed moderate-to-large genetic correlations between the two phenotypes (0.45 and 0.67 at ages 18 and 24, respectively), indicating a substantial overlap in the genetic variants underlying BDD symptoms and suicidality. Non-shared environmental correlations were small (0.17 at both ages 18 and 24), suggesting largely distinct environmental risk factors for BDD symptoms and suicidality.

The path estimates shown in Fig. 1 were used to calculate the proportion of phenotypic association between BDD symptoms and suicidality that was accounted for by genetic and non-shared environmental factors (see Fig. 1 footnote for details). At age 18, genetic influences accounted for 72.9% (95% CIs 48.4–99.0) of the covariance, while non-shared environmental factors accounted for 27.1% (95% CIs 2.0–51.6). Similarly, at age 24 genetic factors explained 77.7% (95% CIs 59.9–94.7) and non-shared environmental factors accounted for 22.3% (95% CIs 5.3–40.1) of the association between BDD symptoms and suicidality. Of note, a similar pattern of results was found when suicidal ideation and suicide attempts were analysed separately at age 24 (see online Supplementary Table S5).

## Discussion

The current study represents the largest investigation of BDD symptoms and suicidality to date and is the first to explore the contribution of genetic and environmental influences to this association. With respect to our first aim, we found that self-reported suicidal ideation and behaviours were significantly elevated among young people with probable BDD, relative to those without. For example, lifetime suicide attempts were self-reported by approximately a quarter of individuals with probable BDD at both ages, compared to around 5% of those without. These figures are similar to the results of a previous meta-analysis, which found the weighted pooled rate of suicide attempts among individuals (mean age = 38 years) with diagnosed BDD to be 24% (Angelakis et al., 2016). By replicating this finding in a community sample, we demonstrate that the association is not only a product of referral or selection bias. Our findings also extend previous research by demonstrating that the strong association between BDD and suicidality is evident in late adolescence. Given that the mean age at onset of BDD has been reported to be 16–17 years (Bjornsson et al., 2013), our findings suggest that suicidality may feature strikingly early in the course of the illness. In the current study, parents reported substantially lower rates of suicide attempts in their children generally (i.e. in both the probable BDD and non-BDD group). Nevertheless, according to parent-report, the risk of suicide attempts was approximately seven times greater in 18-year-olds with probable BDD, compared to those without. Thus, the strong association between BDD and suicidality is robust across informants and not explained by common method variance.

Continuous scores of BDD symptoms were also significantly associated with self- and parent-reported suicidality at ages 18 and 24. This relationship remained significant after controlling for coexisting symptoms of depression and anxiety across all indices of suicidality, with the exception of parent-reported suicide attempts at age 18. Our findings therefore broadly support the notion that BDD symptoms are an independent risk factor for suicidality (Snorrason, Beard, Christensen, Bjornsson, & Björgvinsson, 2019), although further prospective studies are needed to clarify the direction of effects. Our results also imply that aspects of BDD symptomatology beyond the intrinsic features of low mood, depressive cognition, and anxious arousal are related to suicidality. A recent network analysis of BDD and depressive symptoms found suicidal ideation to be particularly linked with time spent on preoccupations and difficulty controlling compulsions (Summers et al., 2020). Future studies should seek to replicate and extend this research, with a focus on suicidal behaviours as well as cognitions.

Our genetic analyses confirm previous findings that BDD and suicidality are both moderately heritable (Althoff et al., 2012; Enander et al., 2018; Fu et al., 2002; Maciejewski et al., 2014; Monzani et al., 2012a; Statham et al., 1998). We also found evidence of substantial genetic overlap between BDD and suicidality, whereas non-shared environmental influences appeared to be largely distinct. This novel finding is consistent with previous research showing substantial genetic pleiotropy across psychiatric traits (Allegrini et al., 2020; Selzam, Coleman, Caspi, Moffitt, & Plomin, 2018). Importantly, in relation to our second aim, we found that genetic influences accounted for the majority of the association between BDD and suicidality (72.9 and 77.7% at ages 18 and 24, respectively). However, non-shared environmental factors also contributed significantly to the covariance (accounting for 27.1 and 22.3% at ages 18 and 24, respectively). Our findings are broadly in line with a recent study that estimated genetic and non-shared environmental factors to account for 60.7% (95% CI 32.1–89.4%) and 40.4% (95% CI 24.2–56.6%) of the coaggregation between OCD and suicide attempts, respectively (Sidorchuk et al., in press). Since OCD and BDD are closely related at both a phenotypic and genetic level (American Psychiatric Association, 2013; Monzani et al., 2012b), it follows that similar mechanisms may underpin their links with suicidality.

The current findings have several important clinical implications. First, they highlight the importance of continuous assessment and careful management of suicidality in individuals with BDD of all ages. We found that approximately a quarter of 18- and 24-year-olds with clinically significant BDD symptoms reported having attempted suicide. Rates may be even higher in clinical settings, where severe and complex cases tend to be overrepresented. Concerningly, our data suggest that parents underestimate suicidality in their adolescents with clinically significant BDD (as well as those without), consistent with previous research demonstrating that parents are often unaware of their child's suicidal behaviours (Breton et al., 2002). In addition, almost a third of young adults with probable BDD who reported having attempted suicide did not seek help from a healthcare professional. Although it is plausible that not all individuals who attempted suicide required medical attention, this finding is also in line with previous studies showing that young people are reluctant to seek help when they are suicidal (Michelmore & Hindley, 2012). Further research is needed to understand barriers to disclosure and help-seeking disclosure in this population, in order to inform education and prevention strategies. Second, our findings suggest that depressive symptoms may not be the only indicator of suicidality among those with BDD symptoms. Specific features of BDD psychopathology appear to be independently related to suicidal thoughts and behaviours, and this should be considered when assessing risk. Third, our results suggest that the majority of the covariance between BDD symptoms and suicidality is accounted for by genetic influences. Thus, identification of genetic variants associated with BDD, for example through genome-wide association studies, could shed light on the biological mechanisms involved in suicidality, and vice versa. Fourth, our findings indicate that non-shared environmental factors also explain a significant proportion of the link between BDD symptoms and suicidality. This is consistent with the clinical notion that suicidality is often a functional consequence of the psychosocial burden of BDD. In other words, non-shared environmental experiences (e.g. failing school examinations, loss of a job, breakdown of a relationship) may mediate a causal pathway between BDD and suicidality. However, our findings could also indicate that BDD and suicidality share some of the same environmental risk factors (e.g. peer victimisation). Further understanding of these environmental factors could help to identify individuals with BDD symptoms who are at high risk for suicidality. Additionally, if these environmental factors are modifiable, they could inform new avenues for prevention and treatment.

Strengths of this study include the utilisation of a relatively large, population-based sample comprising 9481 adolescents and young adults. Furthermore, the age 18 and 24 cohorts were entirely independent, yet we found strikingly similar patterns of results at both ages, providing replication across two developmental stages. Nevertheless, several limitations should be considered. First, cases of probable BDD were identified using a self-report questionnaire, not a clinician-administered diagnostic assessment. It is notable that 2.0 and 1.7% of the sample were classified as having probable BDD at ages 18 and 24, respectively, which is consistent with known BDD prevalence rates (Veale et al., 2016). However, replication and extension of the current findings to diagnosed samples of BDD patients and their biological relatives, as has recently been done in OCD (Sidorchuk et al., in press), would be ideal. Second, our assessment of suicidality relied on single items that varied across the two cohorts, and future studies should incorporate validated assessment measures of suicidal thoughts and behaviours. Third, our analyses focussed on cross-sectional associations and we are therefore unable to determine the direction of effects between BDD symptoms and suicidality. Fourth, there are several limitations that are inherent to twin designs, as previously described, such as not accounting for gene–environment correlations and interactions (Plomin, DeFries, Knopik, & Neiderhiser, 2013; Rijsdijk & Sham, 2002).

In conclusion, the current study demonstrates that BDD symptoms are strongly associated with suicidal ideation and attempts during late adolescence and young adulthood. Importantly, the association appears to be largely underpinned by genetic influences, but non-shared environmental factors are also likely to explain a substantial proportion of the covariance. Identification of these genetic and environmental factors could highlight new opportunities for intervention among high-risk individuals.

## Supplementary material

For supplementary material accompanying this paper visit https://doi.org/10.1017/S0033291720002998. click here to view supplementary material
