# Supplementary material for: The association between body dysmorphic symptoms and suicidality among adolescents and young adults: a genetically informative study
Source: Psychol Med. 2020 Sep 17;52(7):1268–76. doi: 10.1017/S0033291720002998 (PMC9157307; doi:10.1017/S0033291720002998)
Supplement: Supplementary file 1 [file S0033291720002998sup001.docx]

**Table S1: Suicidality items administered at ages 18 and 24.**

|  | **Questionnaire source** | **Item** |
| --- | --- | --- |
| **CATSS-18** | |  |
|  | Adult Behavior Checklist  (parent-report) | [My child . .] Deliberately harms self or attempts suicide |
|  |  | [My child . .] Talks about killing self |
|  | Life History of Aggression  (self-report) | Have you deliberately attempted to kill yourself when you were angry or despondent? |
| **CATSS-24** | | |
|  | Center for Epidemiologic Depression Scale-Revised  (self-report) | [How often have you felt this way in the last two weeks . .] I wished I were dead |
|  | Suicidal Thoughts Questionnaire  (self-report) | Have you ever had thoughts about taking your life? |
|  |  | Have you ever made an attempt to take your life? |
|  |  | If "yes", did the attempt lead to that you went to a doctor, emergency room, or another health care facility? |
|  |  | If "yes", did the attempt lead to that you were hospitalized overnight or longer? |

**Table S2: Skewness of variables.**

|  | **Variable** | **Skewness of raw variable** | **Skewness of log transformed variable** |
| --- | --- | --- | --- |
| **Age 18** |  |  |  |
|  | **DCQ** | 1.53 | .15 |
|  | **CES-D** | .89 | N/A |
|  | **SCARED** | 1.15 | -.94 |
| **Age 24** |  |  |  |
|  | **DCQ** | 1.51 | -.04 |
|  | **HADS-D** | .72 | N/A |
|  | **HADS-A** | .82 | N/A |

Note: DCQ = Dysmorphic Concerns Questionnaire; SCARED = Screen for Child Anxiety Related Emotional Disorders; CES-D = Center for Epidemiologic Studies Depression Scale; HADS = Hospital Anxiety and Depression Scale.

**Table S3: Fit comparisons for bivariate models at age 18.**

| **Base model** | **Comparison model** | **ep** | **-2LL** | **df** | **AIC** | **diffLL** | **diffdf** | ***p*** |
| --- | --- | --- | --- | --- | --- | --- | --- | --- |
| Saturated | N/A | 23 | 21148.19 | 14194 | -7239.81 | N/A | N/A | N/A |
| Saturated | Homogeneity | 11 | 22202.36 | 14208 | -6213.64 | 1054.17 | 14 | 3.71e-216 |
| Saturated | Scalar ACE | 14 | 21169.89 | 14204 | -7238.12 | 21.70 | 10 | .02 |
| Saturated | **Scalar AE** | **11** | **21169.89** | **14207** | **-7244.11** | **21.70** | **13** | **.06** |
| Saturated | Quantitative ACE | 22 | 21152.65 | 14197 | -7241.35 | 4.46 | 3 | .22 |
| Quantitative ACE | Homogeneity | 11 | 22202.36 | 14208 | -6213.64 | 1049.71 | 11 | 3.83e-218 |
| Quantitative ACE | Scalar ACE | 14 | 21169.89 | 14204 | -7238.12 | 17.23 | 7 | .02 |
| Quantitative ACE | Scalar AE | 11 | 21169.89 | 14207 | -7244.11 | 17.23 | 10 | .07 |
| Qualitative ACE (rA = free) | Quantitative ACE | 22 | 21152.65 | 14197 | -7241.35 | 4.12e-09 | 4 | 1.00 |
| Qualitative ACE (rC = free) | Quantitative ACE | 22 | 21152.65 | 14197 | -7241.35 | 4.12e-09 | 4 | 1.00 |
| ScACE | ScAE | 11 | 21169.89 | 14207 | -7244.11 | 8.49e-04 | 3 | 1.00 |

*Note:* ep= estimated parameters; -2LL = minus twice the log likelihood; df = degrees of freedom; AIC = Akaike’s information criterion; diffLL = difference in -2LL; diffdf = difference in df; A = additive genetic influence; C= shared environmental influences; E = non-shared environmental influences. In the saturated model, means, variance, and liability thresholds were equated across twin order and zygosity groups for males and females separately. The qualitative ACE models were not compared to the saturated model because they had more parameters. Qualitative models allowed for quantitative sex differences in addition to qualitative sex differences in genetic (A) or shared environmental (C) influences.

**Table S4: Fit comparisons for bivariate models at age 24.**

| **Base model** | **Comparison model** | **ep** | **-2LL** | **df** | **AIC** | **diffLL** | **diffdf** | ***p*** |
| --- | --- | --- | --- | --- | --- | --- | --- | --- |
| Saturated | N/A | 23 | 12913.86 | 6208 | 497.86 | N/A | N/A | N/A |
| Saturated | Homogeneity | 11 | 13200.41 | 6222 | 756.41 | 286.55 | 14 | 7.5e-53 |
| Saturated | Scalar ACE | 14 | 12933.79 | 6218 | 497.79 | 19.93 | 10 | .03 |
| Saturated | **Scalar AE** | **11** | **12933.79** | **6221** | **491.79** | **19.93** | **13** | **.10** |
| Saturated | Quantitative ACE | 22 | 12918.03 | 6211 | 496.03 | 4.17 | 3 | .24 |
| Quantitative ACE | Homogeneity | 11 | 13175.85 | 6232 | 711.85 | 282.38 | 11 | 4.48-54 |
| Quantitative ACE | Scalar ACE | 14 | 12933.79 | 6218 | 497.79 | 15.76 | 7 | .03 |
| Quantitative ACE | Scalar AE | 11 | 12933.79 | 6221 | 491.79 | 15.76 | 10 | .11 |
| Qualitative ACE (rA = free) | Quantitative ACE | 22 | 12918.03 | 6211 | 496.03 | 2.86e-10 | 4 | 1.00 |
| Qualitative ACE (rC = free) | Quantitative ACE | 22 | 12918.03 | 6211 | 496.03 | 2.86e-10 | 4 | 1.00 |
| ScACE | ScAE | 11 | 12933.79 | 6221 | 491.79 | -2.29e-09 | 3 | 1.00 |

*Note:* ep= estimated parameters; -2LL = minus twice the log likelihood; df = degrees of freedom; AIC = Akaike’s information criterion; diffLL = difference in -2LL; diffdf = difference in df; A = additive genetic influence; C= shared environmental influences; E = non-shared environmental influences. In the saturated model, means, variance, and liability thresholds were equated across twin order and zygosity groups for males and females separately. The qualitative ACE models were not compared to the saturated model because they had more parameters. Qualitative models allowed for quantitative sex differences in addition to qualitative sex differences in genetic (A) or shared environmental (C) influences.

**Table S5: Estimates from bivariate models of BDD symptoms and suicidal ideation/suicide attempts at age 24**

|  | **A** | **E** | **rA** | **rE** | **rPh** | **%A** | **%E** |
| --- | --- | --- | --- | --- | --- | --- | --- |
|  |  |  |  |  |  |  |  |
| BDD symptoms | .43 (.36, .50) | .56 (.50, .63) | .67 (.53, .83) | .15 (.02, .27) | .39 (.34, .43) | 79.5 (61.3, 97.1) | 20.5 (2.9, 38.7) |
| Suicidal ideation | .49 (.35, .60) | .51 (.40, .65) |  |  |  |  |  |
|  |  |  |  |  |  |  |  |
| BDD symptoms | .43 (.36, .50) | .56 (.50, .64) | .83 (-1.00, 1.00) | .13 (-.05, .31) | .33 (.25, .40) | 72.2 (34.7, 1.00) | 27.7 (0.00, 1.00) |
| Suicide attempts | .19 (.03, .52) | .81 (.48, .52) |  |  |  |  |  |

*Note:* BDD = body dysmorphic disorder; A = additive genetic effects; E = non-shared environmental effect; rA = genetic correlation; rE = non-shared environmental correlation; rPh = phenotypic correlation; % A = percentage of phenotypic correlation accounted for by additive genetic factors; % E = percentage of phenotypic correlation accounted for by non-shared environmental factors. 95% confidence intervals in parentheses.
